# The Role of the Gut Microbiota in the Pathogenesis of Parkinson's Disease

**DOI:** 10.3389/fneur.2019.01155

**Published:** 2019-11-06

**Authors:** Dongming Yang, Deming Zhao, Syed Zahid Ali Shah, Wei Wu, Mengyu Lai, Xixi Zhang, Jie Li, Zhiling Guan, Huafen Zhao, Wen Li, Hongli Gao, Xiangmei Zhou, Lifeng Yang

**Affiliations:** ^1^Key Laboratory of Animal Epidemiology and Zoonosis, Ministry of Agriculture, National Animal Transmissible Spongiform Encephalopathy Laboratory, College of Veterinary Medicine, China Agricultural University, Beijing, China; ^2^Department of Pathology, Faculty of Veterinary Sciences, Cholistan University of Veterinary and Animal Sciences, Bahawalpur, Pakistan

**Keywords:** parkinson's disease, gut microbiota, enteric nervous system, gut-brain axis, microbiota-targeted therapies, fecal transplant

## Abstract

It is well-recognized that the gut microbiota (GM) is crucial for gut function, metabolism, and energy cycles. The GM also has effects on neurological outcomes via many mechanisms, such as metabolite production and the gut-brain axis. Emerging evidence has gradually indicated that GM dysbiosis plays a role in several neurological diseases, such as Parkinson's disease (PD), Alzheimer's disease, depression, and multiple sclerosis. Several studies have observed that PD patients generally suffer from gastrointestinal disorders and GM dysbiosis prior to displaying motor symptoms, but the specific link between the GM and PD is not clearly understood. In this review, we aim to summarize what is known regarding the correlation between the GM and PD pathologies, including direct, and indirect evidence.

## Introduction

The gut microbiota (GM), regarded as the “second brain,” is home to ~100 trillion bacteria, as well-fungi and viruses, and is comprised of 10-fold more cells than the human body. Additionally, the total genome of the GM, also known as the gut microbiome, contains ~3 million genes, 150-fold more than the human genome (1). Interestingly, ~1–3 of an individual's GM is common to most people, while the remaining two-thirds is specific to the individual; that is to say, each individual GM is markedly different from others. Notably, 50–60% of the microbes that comprise the GM have never been cultured, as they form resilient spores to facilitate host-to-host transmission (2, 3). Recently, special focus has been placed upon the large fraction of the microbiome that can significantly regulate human behavior along with physical and biological conditions. This work has revealed the existence of a bidirectional network called the “gut microbiota-brain axis” (GMBA). The recognition of the influence of GMBA on the occurrence and mechanism of numerous diseases has ignited the need for further research and new GMBA-based treatment methods (4). The GMBA can influence brain neurochemistry in many ways, including altering the state of neurotransmitters, their receptors, and various other factors, to influence behavior (5–8).

Parkinson's disease (PD), the second most common neurodegenerative disorder after Alzheimer's disease, is a progressive disease that affects the central nervous system and, eventually, the motor system. PD affects an estimated 3 million people worldwide (approximately around age 60), who suffer from often-debilitating motor deficit symptoms, including tremors, bradykinesia, muscle stiffness, and impaired gait (9–11). The key pathological characteristics of PD are the accumulation of the protein alpha-synuclein (α-synuclein [α-syn]), also called Lewy bodies and Lewy neurites, and cell death in the brain's basal ganglia, where up to 70% of the dopamine-secreting neurons in the substantia nigra pars compacta are affected by the end of life (12, 13). This damage to dopaminergic neurons is responsible for the distinctive movement disorder and vagal nerve dysfunction associated with PD. Although PD is commonly regarded as a movement disorder, it has been gradually recognized that PD patients often suffer from some non-motor symptoms, such as rapid eye movement (REM) sleep deficits (14), hyposomia (15, 16), cognitive impairment (17), orthostatic hypotension (18), and most commonly, intestinal dysfunction, with ~80% of PD patients suffering from constipation (19–21). Some of these symptoms may appear several years earlier than the clinical motor symptoms and pathogenesis (Lewy bodies) (22, 23). Studies in rats have demonstrated that α-syn forms spread from the gastrointestinal (GI) tract to the brain, supporting the hypothesis that PD pathogenesis may act primarily via the gut (21, 24–26).

Thus, understanding the interaction between the GM and PD occurrence may open new avenues for PD intervention and therapy. The strength of the evidence supporting this hypothesis has been widely discussed in several excellent recent reviews (27–29) and is briefly summarized below. This review focuses on Braak's hypothesis of PD, the influence of the gut-brain axis, the enteroendocrine cells (EECs)-neural circuit, gut permeability and inflammation, medications and confounders for PD via the GM, fecal microbiota transplant as a treatment for PD, and GM changes in PD to provide a comprehensive overview of the mediatory role of the GM in PD. We also discuss the potential molecular mechanism of the GM and microbial metabolite dysbiosis in PD and GM-targeted interventions for PD.

## The hypothesis of PD in the gut

There was little research on PD pathogenesis prior to 1980. The cause of PD was unclear until the first description of Lewy bodies (LBs). Neurites were quickly recognized in the GI tract of PD subjects as a clinical implication and pathological hallmark of PD (30, 31). However, there was little discussion of LBs in the movement disorder community (32, 33). Only a few researchers have continued to focus on this phenomenon, including Braak et al. who spent more than 20 years contributing to our knowledge of PD pathogenesis in the gut. Braak's observations of PD pathogenesis in the gut suggested that intrinsic and extrinsic innervation of the GI tract, the dorsal motor nucleus of the vagus nerve (DMV), and the enteric nervous system (ENS) were all affected to various degrees during the early progression of PD, even earlier than the substantia nigra. Braak et al. also hypothesized that a kind of unknown neurotropic pathogen may initially damage and destroy innervation of the GI tract and result in Lewy pathology in the gut. Through vagal innervation, LBs in the gut will reach the DMV and eventually move to and damage the substantia nigra, resulting in the appearance of clinical symptoms of PD (34). This model was further supported by observations made of autopsies by Braak et al. who found that LBs and neurites could be observed in the intestinal wall in some PD patients (25). In this postmortem survey, they selected five individuals with increasing severity of CNS LB pathology along with corresponding samples whose brains were devoid of α-syn pathology. After systemically comparing gastric myenteric and submucosal plexuses from these samples, they observed immunoreactive α-syn inclusions similar to those in the DMV in both the gastric myenteric and submucosal plexuses of all five samples with LB pathology. Among these five samples, three had a clinical diagnosis of PD and immunoreactive α-syn inclusions in the substantia nigra. Although the other two cases did not appear to have clinical Parkinsonism, both cases had α-syn pathology in both the stomach and DMV. Moreover, one of these two was even positive in the substantia nigra (35).

Based on previous research, Braak et al. asserted that PD exerted significant effects in the ENS. They observed that involvement of both ENS plexuses began early in PD rather than being confined to the end-stages of the disease, as the lesions could be observed in both clinically diagnosed PD patients and non-symptomatic individuals, suggesting that α-syn pathology may also occur in all of the different innervations of the GI tract in different stages of PD (25, 35). Thus, the process of PD development could be divided into three logical possibilities. (1) An unknown neurotropic PD pathogen may access and damage multiple nervous system sites simultaneously. (2) ENS involvement may be antecedent to brainstem involvement, or brain involvement may precede ENS involvement. (3) There may be an uninterrupted connection between the ENS and CNS that is susceptible to PD pathology, such that this PD pathogen is able to pass through the gastric epithelial lining and induce α-syn pathology via a consecutive series of nerve cell projections, contributing to the diffusion of PD neuropathology from one nerve to the next. Therefore, the clinical pathology of PD could be divided into six different stages. In the first two stages, patients are in the early period of PD and will display some symptoms, such as constipation, insomnia, and impairment of smell, with the appearance of α-syn pathology in the olfactory bulb and DMV. In the third stage, the typical movement-related symptoms, such as tremor, bradykinesia, rigidity, and postural instability, will appear, and the substantia nigra may be positive for immunoreactive α-syn inclusions (36, 37). Eventually, in the last three stages, patients will suffer from the key severe symptoms of PD, such as motor disorders and neuropsychiatric disturbances, as LBs reach the striatum and cerebral cortex.

## Gut-brain axis in PD

**Figure 1:**
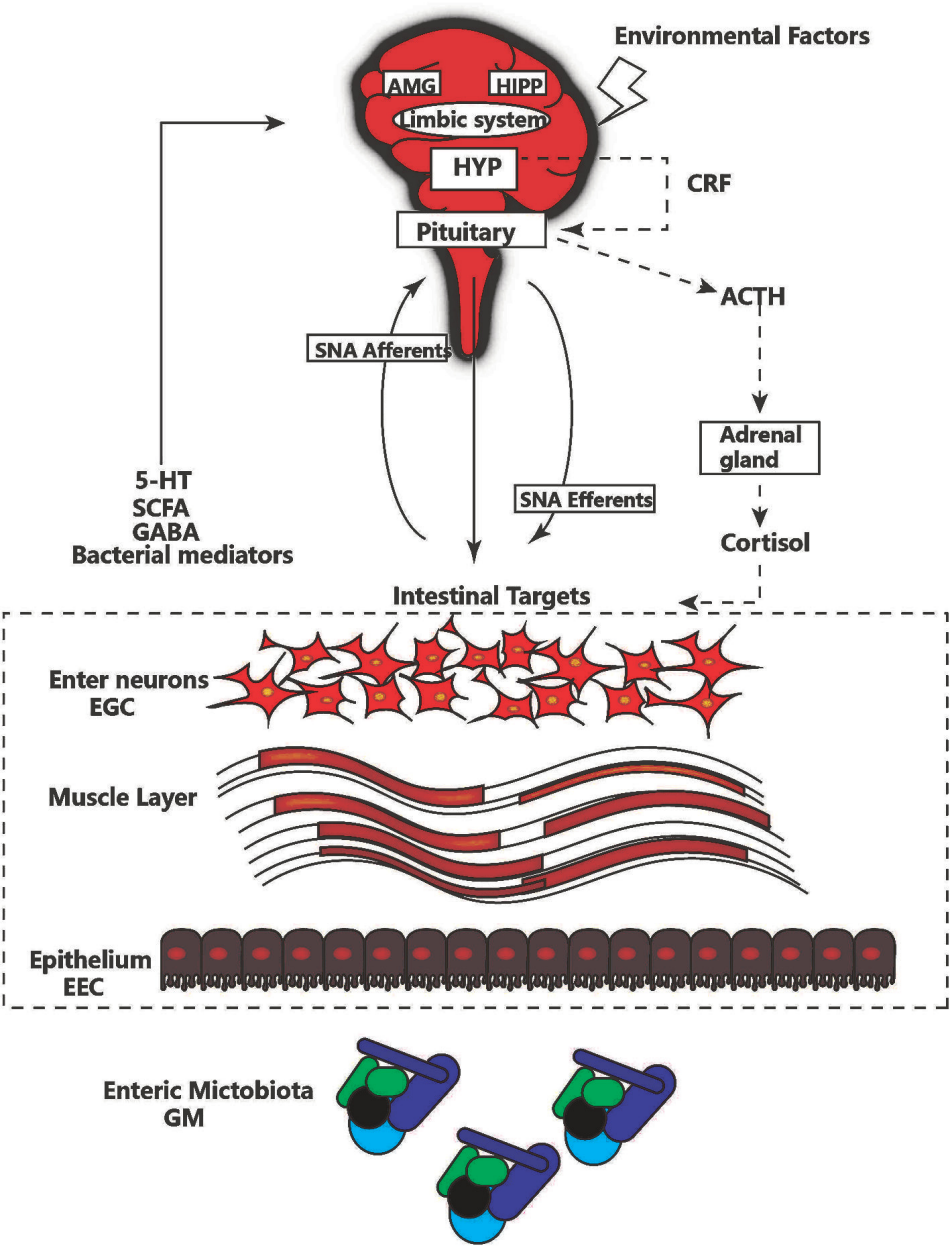
The main components of the gut-brain axis. The gut-brain axis consists of bidirectional communication between the ENS and CNS. The CNS and hypothalamic-pituitary-adrenal (HPA) axis (shown as a dashed line) can be affected by environmental factors, including emotion, and stress. The HPA concludes with cortisol release and is regulated by a complex interaction between the amygdala (AMG), hippocampus (HIPP), and hypothalamus (HYP), which comprise the limbic system. HYP secretion of corticotropin-releasing factor (CRF) stimulates adrenocorticotropic hormone (ACTH) secretion from the pituitary gland, which in turn leads to cortisol release from the adrenal gland. In parallel, the CNS communicates with intestinal targets through both afferent and efferent autonomic pathways (SNA). Diverse factors from different parts of the GI tract, including the GM, enteric neurons, and enteric glial cells (EGG), interact with 5-hydroxytryptamine (5-HT), short-chain fatty acids (SCFAs), and neurotransmitters (GABA) to affect the CNS, resulting in bidirectional communication.

**Figure 2:**
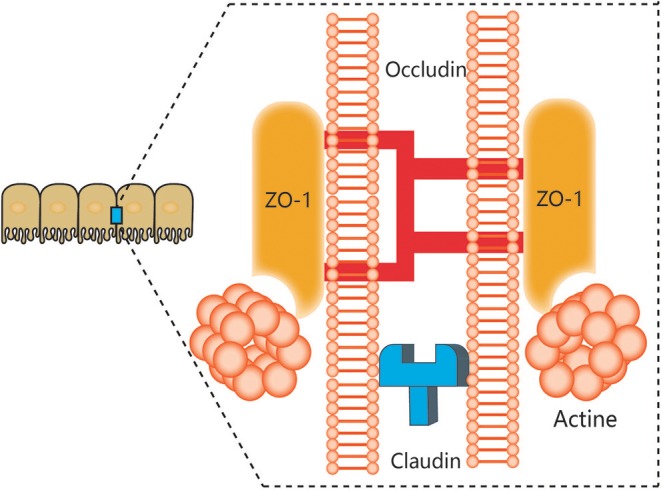
Components of tight junctions. Tight junctions (TJs) of epithelial intestinal cells form selective barriers that regulate paracellular permeability. The main proteins that compose TJs include zonula occludens-1 (ZO-1), claudins, and occludin.

The notion of a gut-brain axis (GBA) was initially proposed by Sudo et al. in 2004 when they observed an impaired stress response in germ-free mice (38). The GBA is the bidirectional communication between the CNS and the ENS that connects the emotional and cognitive centers of the brain to the peripheral intestinal functions provided by the endocrine and immune systems, the intestinal epithelium, and the GM (Figure 1). It includes several nervous system components, such as the CNS, the brain and spinal cord, the autonomic nervous system, the ENS, and the hypothalamic-pituitary-adrenal (HPA) axis. The autonomic system, including the sympathetic and parasympathetic limbs, drives both afferent signals arising from the lumen and transmitted through enteric, spinal, and vagal pathways to the CNS and efferent signals from the CNS to the ENS and intestinal wall (39, 40). The vagus nerve, which innervates the entire intestinal tract to the left colonic flexure, is considered the sensor of microbiota metabolites and transfers this information to the brain (39). The HPA axis is part of the limbic system, which is involved in memory and emotional responses and functions as the core stress efferent axis coordinating adaptive responses to any kind of stressor (41). Environmental stress or systemic pro-inflammatory signals can activate the limbic system through secretion of corticotropin-releasing factor (CRF) by the hypothalamus; in turn, this stimulates secretion of adrenocorticotropic hormone (ACTH) from the pituitary gland and finally leads to cortisol release from the adrenal glands. Cortisol is a major stress hormone that is involved in many metabolic reactions, including those in the brain. The ENS is an integrative neuronal network of two ganglionated plexuses, myenteric and submucosal, and is composed of neurons and enteric glial cells (EGCs) (42). It has been suggested that EGCs represent the ENS counterpart of CNS astrocytes, as they are similar to astrocytes both morphologically and immunohistochemically (43). The humoral components of the gut-brain axis consist of the enteroendocrine system, the mucosal immune system, and microbiota-derived metabolites. Enteroendocrine cells (EECs) can produce hormones such as ghrelin and 5-hydroxytryptamine (5-HT), which have a wide range of effects on gut and brain functions (44). The intestinal epithelium forms a regulated barrier, known as the intestinal epithelium barrier (IEB), between the circulating blood and the contents of the intestinal lumen, and functions to prevent the passage of outer noxious pathogens and to absorb and secrete nutrients (45). Among the structures in the IEB, the epithelial tight junctions are the most important, as they connect adjacent enterocytes together to determine the paracellular permeability through the lateral intercellular space (Figure 2). These junctions are composed of transmembrane proteins such as claudins and occludins connected to the actin cytoskeleton via high molecular weight proteins called zona occludens (46). These structures are under the influence of the GM and its metabolites, which play a vital role in reciprocal gut-brain communication (47). All of the elements of the gut-brain axis described above may be individually affected by PD pathology to various degrees.

## Enteric neuron damage in PD

The α-syn protein is generally expressed in the central nervous system (CNS), mainly in presynaptic terminals. Studies suggest that it plays a role in modulating the supply and release of dopamine to regulate neurotransmission. The main pathological characteristic of PD is the accumulation of α-syn in the form of Lewy bodies in cell somata and Lewy neurites in axons and dendrites.

According to Braak's hypothesis, the innervation of the GI tract is the most sensitive and easily damaged. The ENS injury caused by the unknown PD pathogen may present as α-syn pathology, and several clinical studies revealed that PD patients displayed α-syn accumulation in the ENS. Accumulation of α-syn is related to damage of enteric neurons and is possibly associated with GI tract dysfunction. This type of protein accumulation affects both the myenteric and submucosal plexuses of the gut in PD patients and is gradually distributed from the most distal point of the esophagus to the rectum. Braak and his colleagues hypothesized that α-syn accumulation may follow the path of the vagal nerve to spread from the ENS to the brain through the brainstem, midbrain, basal forebrain, and finally, the cortical areas (25, 35–37). Recent studies demonstrated that gut-initiated pathological processes in PD can be directly caused by GM disorder, not only by a PD pathogen or environmental toxin. These disordered microorganisms may initiate α-syn accumulation in either the olfactory bulb or the enteric nerve cell plexus, causing concurrent mucosal inflammation, and oxidative stress (48, 49). Pan-Montoji observed that intragastric administration of the pesticide rotenone, an inhibitor of mitochondrial complex I activity, can induce PD-like neuropathological changes that can be seen in the ENS and later in the substantial nigra pars compacta (50). Furthermore, other researchers found that this phenomenon was prevented by hemivagotomy or resection of the autonomic nerves (51). Holmqvist made similar observations accordant with this result and consistent with Braak's hypothesis of the early appearance of LBs in neurons projecting from the vagus nerve in PD. He found that α-syn can be retrogradely transported from the intestinal wall to the brain following the injection of monomeric or oligomeric α-syn into the intestinal wall in rats (52).

Others have shown, using *in vitro* and *in vivo* experiments, that α-syn can be transmitted via endocytosis to neighboring neurons. In recent studies, full truncal vagotomy was found to reduce the risk of developing PD when compared with highly selective vagotomy, which only affects the acid-producing portion of the gastric body, or with no vagotomy (53). These results suggest that the vagal nerve might provide a path for the spread of PD from the gut to the brain. The presence of gut LB pathology can be traced in living subjects through biopsy. Multiple studies assessed GI tissue to check α-syn pathology by endoscopy. Shannon identified three samples with α-syn deposition in sigmoid colon biopsies prior to the appearance of typical PD symptoms. In a subsequent study, seven of 62 patients displayed α-syn accumulation in gastric, duodenal, and colonic biopsies more than 8 years prior to the onset of the motor symptoms of PD (26). More recently, Stokholm selected 39 PD patients with an average of 7 years prior to the onset of motor symptoms and analyzed various regions of the GI tract via paraffin-embedded tissue blocks. Of these 39 patients, 22 were found to have phosphorylated α-syn depositions (54).

**Figure 3:**
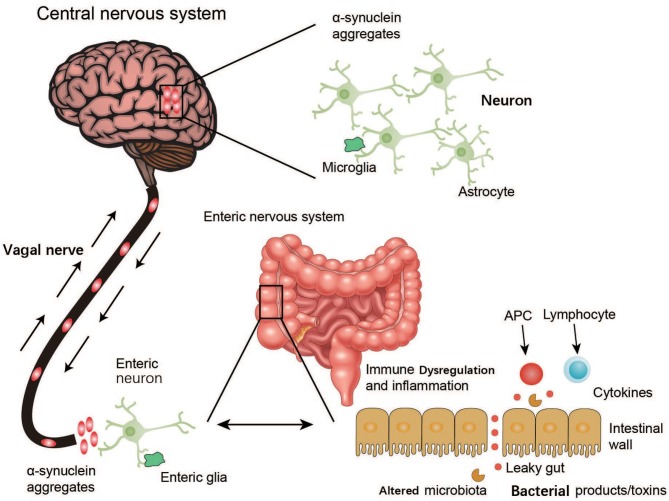
Schematic representation of α-synuclein accumulation and aggravation from the ENS to the brain. Environmental factors like microorganisms (including the GM) and unknown pathogens may initiate a pathological process in the enteric nerve cell plexus, leading to mucosal inflammation, and oxidative stress and further α-synuclein accumulation. The vagal nerve may act as a path for the spread of α-synuclein pathology from the ENS to the brain through the brainstem, midbrain, basal forebrain, and finally, the cortical areas.

The findings of the above studies suggest that the ENS is one of the initial sites of α-syn accumulation and may be connected with the PD pathogen. However, how this PD agent causes changes in the ENS through oral administration is unknown, as only enteric nerves in the submucosa of the intestine, and not the intestinal lumen, are affected. Although the gut may be the location where PD arises, how this ingested PD pathogen triggers α-syn pathology within enteric neurons remains to be explored (Figure 3).

## The EECs-neural circuit

Enteroendocrine cells (EECs) are the specialized endocrine function cells that reside in most of the mucosa of the GI tract. Recent research has found that EECs, as body endocrine cells, also possess electrical excitability and chemosensitive properties to allow for the production of gastrointestinal hormones or peptides in response to various stimuli and their release into the bloodstream to exert systemic effects. It is believed that the anatomical positions of EECs, in the apical surface exposed to the gut lumen and the basal portion where various secretory granules are contained, allow EECs to respond to signals from bacteria and pathogens in the gut. Recently, EECs were found to possess not only neuron-like features like neurotrophin receptors, pre- and post-synaptic proteins, and neuropods, but also the enzymatic machinery required for dopamine synthesis (55).

The expression of synaptic proteins makes it possible for EECs to make contact with nerves, as demonstrated by using a retrograde rabies virus tracing technique. Thus, EECs are a newly recognized neural circuit that connects the nervous system with the gut lumen, which raises a number of interesting possibilities (56). First, EECs may act as a portal for the entry of pathogens to invade the nervous system, which is affected by distinct kinds of pathogens. Second, EECs can be the first to receive stimuli from the gut lumen, generate a response to those signals, and transform and eventually send those messages to the brain via enteric nerves and cause the release of hormones or neurotransmitters (57). Third, given their neuron-like properties, EECs may easily display neuron-type abnormalities, as supported by many recent discoveries of neuron-like α-syn expression in EECs. Due to their location at the interface of the gut lumen and enteric nerves, EECs may be subject to pathogen or toxin exposure, which could affect α-syn accumulation (57). It is possible that misfolding of α-syn in EECs and transmission to enteric neurons are the first steps in a prion-like cascade that culminates in PD.

## GM-related gut permeability and inflammatory mediators in PD

The gut epithelium acts as a barrier against invasion by pathogens (58). Disruption of gastrointestinal barriers can lead to a series of positive feedback loops that significantly alter the GM to favor inflammophiles, intestinal inflammation, and reactive oxygen/nitrogen species in the gut lumen (59, 60). Thus, destabilized gastrointestinal barriers can be an effective means used by the GM to alter the GMBA and for the translocation of bacteria and their products. This results in increased mucosal permeability, oxidative stress, and inflammatory reactions, along with aggregation of α-syn in the ENS (61, 62).

Intestinal permeability or “gut leakiness” has often been found to be increased in PD patients compared with healthy counterparts and in mouse models of PD (63) and is accompanied by increased deposition of α-syn in the ENS and with tissue oxidative stress. However, a few studies have demonstrated morphological changes of the intestinal epithelial barrier but no changes in the permeability of the gut barrier. Moreover, several studies indicated that patients with inflammatory bowel disease, which is known to increase intestinal permeability, have increased risk of PD, which further suggests a role for gastrointestinal inflammation in the development of PD (58). Studies have also shown that the gastrointestinal innate immune system activated by the GM can strengthen the inflammatory response to α-syn (64).

Various studies have reported that inflammation, which is caused by specific microbial cell structures and pattern-inflammatory recognition receptor signaling pathways, may be closely involved in PD. Several studies on inflammation in the ENS suggested that increased abundance of *Escherichia coli* (36) and the Proteobacteria *Ralstonia* (65) may cause intestinal inflammation and reflect high exposure to endotoxins, including lipopolysaccharide-binding protein, along with increased levels of pro-inflammatory cytokines, such as TNF-α, IFN-gamma, IL-6, and IL-1β, and activation of EGCs (66, 67). By investigating inflammation in the CNS in PD, researchers found evidence of increased inflammatory cytokines, including IL-6 and IL-1β, and expression of the glial cell marker glial fibrillary acidic protein (GFAP). These findings support the hypothesis that the GMBA is involved in PD. At the genetic level, single nucleotide polymorphisms in the *CARD15* gene, which is associated with Crohn's disease, were found to be over-represented in PD patients (68). Moreover, the leucine-rich repeat kinase 2 (*LRRK2*) gene was found to be the most highly associated gene with both familial and sporadic PD and is recognized as the major susceptibility locus for PD (69, 70). The mRNA expression levels of all pro-inflammatory cytokines were significantly unregulated in the ascending colon of PD patients, as assessed using real-time PCR to analyze colonic biopsies from 19 PD patients (67). However, the levels of proinflammatory cytokine expression were significantly heterogeneous among PD patients, with some subjects showing similar levels to control subjects and others exhibiting 4–6-fold upregulation compared with control groups (67). Another study observed increased expression of GFAP along with increased mRNA expression of pro-inflammatory cytokines using qPCR and Western blot (71). Moreover, TNF-α, IL-1β, and GFAP were upregulated in the colon when 6-hydroxydopamine was injected into the medial forebrain bundle of a rat PD model (72). Recent studies also found elevated levels of IL-1α and−1β and C-reactive protein in the feces of PD patients using a multiplex immunoassay, which also demonstrated correlations of these protein levels with age and duration of PD (73). According to the current literature, mediators of inflammation in the CNS and ENS in PD can be divided into four possible mechanisms. [1] CNS lymphatics: a model proposed by Louveau in which the dural sinuses transmit both fluid and immune cells from the cerebrospinal fluid, indicating a possible pathway between the GI tract and the CNS (74). [2] Interaction between Toll-like receptors and α-syn: this interaction may activate microglial responses, aggravate the deposition of α-syn, and result in increased dopaminergic degeneration in the substantia nigra (75). [3] Molecular mimicry: a mechanism where foreign antigens share nucleotide sequences and/or structural similarities with self-antigens (76). The molecular mechanism in PD may originate in the GM, where the production of extracellular amyloid antigens can lead to activation of the innate immune system (77). [4] Inflammaging: the notion that aging is related to increased low-grade chronic inflammation, which commonly appears due to latent infections with viruses along with increased levels of inflammatory markers, such as TNF-α, IL-6, and C-reactive protein. It is associated with aging and alteration of the GM (78).

Altogether, all of the above results demonstrate that the GM is associated with and involved in gut permeability and the inflammatory response in PD patients. Although there have been no consistent conclusions regarding the specific effect of GM alterations in gut permeability and GI inflammation in PD, these findings are consistent with observations that the GM may be directly or indirectly involved in gut permeability and inflammatory responses in the GI and CNS in PD and hence may affect PD pathology.

## GM changes in PD

Clinical research has revealed that more than 80% of patients with PD suffer from various severe GI symptoms such as constipation, nausea, and vomiting, as well as increased intestinal permeability, also known as leaky gut. These symptoms may reflect GM disorder in PD patients (36) and are well-correlated with the intestinal α-syn pathology, ENS neurodegeneration, local inflammation, and oxidative stress observed in PD patients (67, 79, 80).

Several studies have focused on describing the changes in GM composition in PD and altering the GM in patients suffering from PD. One of the most extensive studies demonstrated an association of PD with the bacterium *Helicobacter pylori*. Among PD patients, there is high infection by *H. pylori*, which hinders the absorption of levodopa, a primary drug for treatment of PD in patients with motor impairments (81). Similarly, small intestinal bacterial overgrowth (SIBO), a disorder characterized by excessive bacterial growth in the small intestine, was also found to be associated with PD. Nearly one-quarter of PD patients suffer from SIBO, a rate dramatically greater than that observed in healthy controls. SIBO is related to motor impairments, and its eradiation leads to improvement of motor symptoms, which can be further attributed to peripheral factors, including abnormal absorption of drugs in the GI tract and SIBO-associated malabsorption due to alteration of the composition of chyme (82, 82–84). Furthermore, SIBO may also impair absorption due to associated inflammation in intestinal mucosa or altered metabolism by intraluminal bacteria (82). In a recent study, Scheperjan et al. observed a significant reduction in *Prevotellaceae* in fecal samples of PD patients compared to the control group, which resulted in GM dysbiosis. Upon further study, they found that the relative abundance of *Enterobacteriaceae* was positively correlated with the severity of postural instability and gait difficulty in PD patients (84). Further findings supporting gut dysbiosis in PD pathogenesis include the observation that PD patients exhibit lower levels of *Prevotella, Lactobacillus, Peptostreptococcus*, and *Butyricicoccus* spp. and increased levels of *Proteus* and *Enterobacter* spp. compared with healthy controls (85). PD patients at different stages of the disease appear to display different GM alterations. High levels of *Clostridium coccoides* and *Lactobacillus gasseri* were associated with early PD and advanced PD, respectively (66). A recent study based on 197 cases of PD with 130 healthy controls found that the relative abundances of *Bifidobacteriaceae, Christensenellaceae, Tissierellaceae, Lachnospiraceae, Lactobacillaceae, Pasteurellaceae*, and *Verrucomicrobiaceae* were significantly altered in PD (86). Arumugam et al. also found that under-representation of *Prevotellaceae* decreases the levels of health-promoting neuroactive short-chain fatty acids (SCFAs) as well as thiamine and folate biosynthesis capacity, an observation that is consistent with the decreased levels of these vitamins observed in PD patients (87). *Prevotella* may be associated with a reduction in mucin synthesis, which is associated with increased gut permeability and may enhance the translocation of bacterial antigens (88). In addition to a decreased abundance of *Prevotella*, PD patients exhibit an increased abundance of *Lactobacilliceae*. These changes may be related to lower levels of ghrelin, a gut hormone whose secretion is impaired in PD patients and that may be involved in maintaining and protecting the normal function of nigrostriatal dopamine (88, 89). Keshavarzian et al. investigated differences between the mucosal and fecal microbial communities of PD patients and those of healthy subjects. They were found to be similar, with some notable clinical phenomena in PD patients due to increased mucosal permeability and systemic endotoxin exposure from coliform bacteria (65). They identified *Blautia, Coprococcus*, and *Roseburia* as having lower abundances in PD fecal samples; these SCFA butyrate-producing bacteria belong to genera associated with anti-inflammatory properties. Their reduction may cause a decrease in SCFA levels and, eventually, gut leakiness. Additionally, genes involved in lipopolysaccharide biosynthesis and type III bacterial secretion systems were found to be higher in the fecal samples of PD patients compared to those of healthy controls. Type III bacterial secretion systems are generally associated with pathogenicity and translocation of proteins, which could aggravate bacterial-induced inflammation (90, 91). In a recent study, Unger et al. showed that both GM and fecal SCFA concentrations were significantly reduced in PD patients when compared with those from age-matched healthy controls (92). They also found significant reductions in acetate, propionate, and butyrate in PD fecal samples; according to previous studies, SCFA butyrate exerts anti-inflammatory action via an epigenetic mechanism or activation of SCFA receptors, resulting in anti-inflammatory effects, anti-microbial activity, and decreased intestinal barrier leakiness. Thus, altered SFCA abundance may lead to changes in the ENS and contribute to GI dysmotility in PD (7, 93–95). In another study, bacteria from the genus *Faecalibacterium* were found to be significantly reduced in the mucosa of PD samples, while bacteria from the genus *Ralstonia* were significantly increased. No change in the abundance of *Bifidobacteria* was observed in PD patients (65, 96). Using the Movement Disorder Society-United Parkinson's Disease Rating Scale scoring system, low counts of *B. fragilis* and *Bifidobacterium* were found to be associated with worsening of motivation/initiative and hallucinations/delusions, respectively (97). Gut microbial interventions for PD are supported by the fact that enzymes involved in dopamine synthesis in the brain are controlled by the GM via the GMBA (5, 98). Additionally, GM such as *Bacillus* spp. were found to produce dopamine, and almost half of the body's dopamine production is produced by the GM (5). Therefore, PD pathogenesis may be caused or exacerbated by GM disorder and microbiota-induced inflammatory responses. This may promote α-syn pathology from the intestine to the brain or through a rostral-to-caudal route of transfer from cell to cell caused by increased oxidative stress, which may be due to the increase in pro-inflammatory bacteria (99, 100).

It is currently difficult to determine if the observed GM changes are a cause or an effect of PD. However, GM changes may play a key role in neuronal loss through the promotion of inflammatory cascades and oxidative stress in the brain via SCFA-production or a lipopolysaccharide (LPS)-mediated mechanism.

## Medications and confounders for PD via the GM

The current popular anti-Parkinsonian therapeutic strategy is to compensate for dopaminergic cell loss and enhance dopaminergic neurotransmission by using dopamine receptor agonists and the dopamine precursor L-3,4-dihydroxyphenylalanine (levodopa). Oral substitution of levodopa is most effective in curing PD so far; 85% of PD patients between 2001 and 2002 were treated with levodopa (101). However, levodopa treatment cannot stop disease progression and some major symptoms; non-motor symptom patients are non-responsive to levodopa, and its prolonged use may cause severe side effects, such as dyskinesia, motor fluctuations, and even levodopa resistance (102). Further research found that levodopa-unresponsive features and constipation were positively correlated with the severity of α-syn in the ENS (103, 104). In addition, oral treatment with levodopa requires good GI tract function to ensure optimal drug metabolism, which can cause delayed gastric emptying in healthy volunteers and exacerbate GI symptoms in PD patients in clinical settings (105).

**Figure 4:**
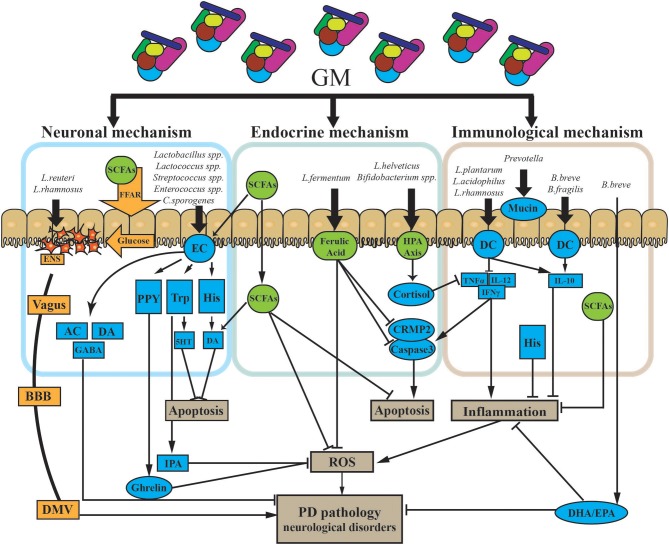
Potential mechanism of probiotic treatment in PD. The GM impacts PD via three primary modalities (neuronal mechanism, endocrine mechanism, and immunological mechanism). [1] The GM can both produce and stimulate certain neurotransmitters via/or not via secretory ECs. ECs can also produce certain neuroactive factors, such as PYY, Trp, and His. These two types of components cross into the BBB and impact the CNS. Some gut hormones stimulated by neuroactive components, like ghrelin and IPA, can have dual effects on the CNS. The GM can directly trigger electrical signals in the ENS through the vagus nerve to the DMV. Finally, the GM may release glucose through SCFAs and FFA to propagate signals through the ENS. [2] The GM can directly and indirectly affect a battery of endocrine signaling components. SCFAs, as the main microbial metabolites, are major signaling molecules that may activate several pathways, as shown in the figure. HPA axis stimulation and the release of endocrine components are also triggered by the GM. Those endocrine components, including cortisol, and ferulic acid, have multiple roles in several pathways in PD. [3] Specific GM members could suppress both chronic and pathological inflammation. Microbe-associated molecular patterns on the surface of GM members directly activate receptors on immune cells like DCs and upregulate/suppress inflammatory cytokines. Finally, the GM influences the production of mucin through the gut. AC, acetylcholine; BBB, blood–brain barrier; CNS, central nervous system; CRMP2, collapsin response mediator protein family; DA, dopamine; DHA, docosahexaenoic acid; DMV, dorsal motor nucleus of the vagus; EC, enterochromaffin cell; ENS, enteric nervous system; EPA, eicosapentaenoic acid; FFAR, free fatty acid receptors; GABA, gamma aminobutyric acid; GM, gut microbiota; His, histamine; HPA axis, hypothalamic–pituitary–adrenal axis; IFNγ, interferon gamma; IL-10, interleukin 10; IL-12, interleukin 12; IPA, indole-3-propionic acid; PYY, peptide YY; ROS, reactive oxygen species; SCFAs, short-chain fatty acids; TNFα, tumor necrosis factor alpha; Trp, tryptophan; 5HT, serotonin.

Although there are no therapeutic strategies that can stop PD progression by directly targeting the GMBA, dietary interventions may influence both the GMBA by shaping the GM and the neuronal functions of the ENS and CNS to improve PD pathogenesis. [1] Nutritional membrane precursors and cofactors: several studies found that specific precursors and cofactors may alleviate synaptic loss and membrane-related ENS and CNS pathology in PD and also reduce motor and non-motor symptoms in preclinical studies. Combination with prebiotic fibers may add therapeutic value for treatment (106). [2] Probiotics: specific probiotics were shown to restore the GM and maintain immune homeostasis. Commonly used probiotics include *Lactobacilli, Enterococci, Bifidobacteria*, yeasts, and mixtures of different beneficial bacteria (107, 108). Recent evidence supports that probiotics can also modulate brain function by improving anxiety and depression (109). Probiotics may be a powerful tool to alter the composition of the GM and improve GI function and associated neuro-inflammation and even levodopa absorption in PD (110). The specific mechanisms associated with probiotics are outlined in Figure 4. [3] Prebiotics: prebiotics are non-digestible ingredients that benefit the host by stimulating/limiting the number of specific GM in different categories (111). Two well-known prebiotics are galacto-oligosaccharides (GOS) and fructo-oligosaccharides (FOS), which are metabolized by most of the *Bifidobacteria* in the colon to produce metabolites like SCFAs, lactose, hydrogen, and methane to antagonize proliferation of pathogenic bacteria (65). Although there have been no investigations of prebiotics for PD patients, evidence supports the use of prebiotics in treating GI dysfunction and improving immune function and neuroprotection. Notably, SCFAs are essential for intestinal epithelial integrity and mucosal immunological responses. Moreover, SCFAs can also activate microglial signaling and affect the expression of T-regulatory cells to increase the level of cytokines to regulate neuro-inflammatory pathways (85). As mentioned above, prebiotics may be a candidate approach to correct the low abundance of SCFAs in PD patients (92).

There is emerging evidence to support the hypothesis that lifestyle differences can also contribute to PD pathology. Smoking and coffee consumption may reduce the possibility of PD, an effect that was hypothesized to be mediated via the GM (112). Studies have suggested that the beneficial effects of coffee consumption and smoking may be associated with the GMBA through alteration of the GM and mitigation of intestinal inflammation (96, 113). Further studies also found that red wine and tea could have a similar effect to coffee to reduce PD predisposition (114). Stress may also be involved in PD, as stress exacerbates intestinal inflammation, gut permeability, endotoxemia, neuroinflammation, and dopamine loss in a PD mouse model (115). A daily diet with increased plant carbohydrates and fiber was found to increase some specific macronutrients deficient in PD patients (87). A western diet is known to contain high amounts of refined carbohydrates and saturated fats, high-fat foods, and whole dairy products that could cause GM dysbiosis and be involved in the process of PD (116, 117). Recent studies found that exercise could also influence the GM along with energy homeostasis and regulation, which may benefit host metabolism and the gut environment (118, 119). Additionally, antibiotics and microbial toxins produced by the GM, including lipopolysaccharide and epoxomicin, could induce significant changes in the GM and the inflammatory response and have effects on neurological diseases via GMBA interactions (120, 121).

## Fecal microbiota transplants for PD

Fecal microbiota transplantation (FMT), also known as stool transplant, is an emerging technique involving the transplant of fecal bacteria from a healthy donor into the GI tract of a recipient. As early as 1,700 years ago, FMT was proposed and applied in traditional Chinese medicine as a treatment for human GI diseases (122). Currently, although there are several methods to restore and modulate the GM including the use of antibiotics and probiotics, FMT remains a comprehensive method to restore the GM ecosystem. FMT generally involves several steps, including screening for specific pathogens, followed by homogenization, filtration, and resuspension of the fecal sample and then delivery by colonoscopy, enema, orogastric tube, or by mouth in the form of a capsule containing freeze-dried material (78). The goal of FMT is to restore healthy gut microbiota components, and it has gained increasing prominence as a treatment modality, with some experts calling for it to become a key therapy for various diseases such as *Clostridium difficile* infection, ulcerative colitis, type 2 diabetes, and neurodegenerative disorders like PD (79, 80). Patients with neurodegenerative disorders often suffer from changes in GI tract motility. For instance, chronic or idiopathic constipation is generally found in PD patients as a co-morbidity and is related to colonic and anorectal dysmotility. Several studies suggest that FMT is beneficial for treating constipation in PD and also results in noticeable improvement of non-GI symptoms in patients with neurological disorders. The discovery of the mechanisms underlying GM modulation of PD pathogenesis has been given high priority in current research. Proposed methods to evaluate FMT as a potential treatment in PD mainly assess direct communication through the vagus nerve, changes in neurotransmitter metabolites, immune response activation, and production of neuroactive metabolites and neurotoxins (5, 35, 123, 124). As most PD patients suffer from many GI-related symptoms, the autonomic nervous system (ANS), which connects the gut and brain together with the vagus nerve system to relay signals in the GMBA, has become a novel enquiry tool. Autonomic ANS input from the gut also connects to the limbic system of the brain, which is comprised of the amygdala, the hippocampus, and the limbic cortex. Among those parts, the limbic cortex, which has the most essential function, regulates the motor functions that are impaired in PD. The link between the ANS and the limbic system strongly suggests a relationship between gut health and the brain and behavior (125). A recent study using a mouse model of PD as the recipient found that fecal transplant from PD patients exacerbated motor impairment and was associated with a reduction in *Lachnospiraceae* and *Ruminococceae*, the same genera that were dramatically reduced in fecal samples from PD patients (65). In addition, FMT from PD patients, compared with that from healthy controls, could aggravate α-syn–related motor dysfunction in α-syn–overexpressing mice (85). Further studies found that FMT can protect MPTP-induced PD mice by reducing the activation of microglia and astrocytes in the substantia nigra, along with decreasing expression of TLR4/TNF-α signaling pathway components in the gut and brain (126). These findings all support the GM being involved in PD. Several clinical cases of patients with PD, Alzheimer's disease, chronic fatigue syndrome, multiple sclerosis, and other neurological disorders have shown that FMT and/or antibiotic treatment resulted in remission of symptoms in the treatment of gastrointestinal tract (GIT) co-morbid with PD, including constipation, bowel disorders, and ulcerative colitis. FMT has fewer side effects than the traditional chemical PD treatments like levodopa, dopamine agonists, and monoamine oxidase B (MAO-B) inhibitors. Further, FMT therapy may also be helpful in relieving several non-GIT comorbid disorders and may provide additional support for the association between the GM and PD (124, 127, 128).

## Conclusion

Given the research on the connection between the GM and PD to date, the most certain conclusion is that GM disorder in PD patients exacerbates α-syn deposition in PD via many mechanisms and will aggravate neurodegeneration, and thus, PD-related symptoms, such as movement disorders. However, questions and doubt remain in several areas. The exact nature of the relationship of GM disorder with PD remains unclear. Current evidence indicates that the unknown outside pathogen that eventually leads to PD first moves into the GI tract and results in GM imbalance and gradually breaks the intestinal epithelial barrier to reach the ENS. α-syn deposition in PD may then start in the ENS, accumulate to a certain threshold, and finally propagate to the CNS via trans-synaptic cell-to-cell transmission. GM translocation caused by the pathogen may also induce a pro-inflammatory environment in the GI tract. Those signals would be systemically sent to a specific part of the brain through dysfunctional blood-brain barrier structures. Studies found that α-syn was present in both the gut and brain (129, 130), which suggests that the gut may not be the first point of α-syn pathology in PD. Thus, we cannot yet draw firm conclusions on the digestive origin of PD. Further studies aimed at the GMBA and the impacts of manipulating the GM and microbial metabolites on PD are needed to establish a cause-and-effect relationship between GM dysbiosis and PD. Although research in this area is still preliminary, the mechanism underlying GM influence and regulation of the CNS in PD may be explored in greater detail in the future, as the GM has been found to regulate microglia and mediate neurophysiological processes at several levels.

Although no treatment to cure PD completely has yet been designed, an improved understanding of the interaction of the GM and the brain may shed new light on the pathological progression of PD and provide new therapeutic possibilities. For example, FMT and the discovery of new GI biomarkers for clinical diagnosis of PD may represent a new avenue of PD treatment distinct from traditional chemical treatments like levodopa (101).

## Author Contributions

DY wrote the first draft of the manuscript. DZ and LY revised the initial drafts and provided scientific contributions. All remaining authors edited the final version of the manuscript.

### Conflict of Interest

The authors declare that the research was conducted in the absence of any commercial or financial relationships that could be construed as a potential conflict of interest.

## References

[1] Franzosa EA Katherine H Meadow JF Dirk G Lemon KP Bohannan BJM. Identifying personal microbiomes using metagenomic codes. Proc Natl Acad Sci USA. (2015) 112:E2930. 10.1073/pnas.1423854112 25964341 PMC4460507

[2] Walker AW Duncan SH Louis P Flint HJ. Phylogeny, culturing, and metagenomics of the human gut microbiota. Trends Microbiol. (2014) 22:267–74. 10.1016/j.tim.2014.03.001 24698744

[3] Browne HP Forster SC Anonye BO Kumar N Neville BA Stares MD. Culturing of ‘unculturable' human microbiota reveals novel taxa and extensive sporulation. Nature. (2016) 533:543–6. 10.1038/nature17645 27144353 PMC4890681

[4] Nicholson JK Elaine H James K Remy B Glenn G Wei J. Host-gut microbiota metabolic interactions. Science. (2012) 336:1262–7. 10.1126/science.1223813 22674330

[5] Cryan JF Dinan TG. Mind-altering microorganisms: the impact of the gut microbiota on brain and behaviour. Nat Rev Neurosci. (2012) 13:701–12. 10.1038/nrn3346 22968153

[6] Rochellys DH Shugui W Farhana A Yu Q Britta BR Annika S Normal gut microbiota modulates brain development and behavior. Proc Natl Acad Sci USA. (2011) 108:3047–52. 10.1073/pnas.1010529108 21282636 PMC3041077

[7] Forsythe P Kunze WA. Voices from within: gut microbes and the CNS. Cell Mol Life Sci. (2013) 70:55–69. 10.1007/s00018-012-1028-z 22638926 PMC11113561

[8] Premysl B Emmanuel D Josh C Wendy J Jun L Jennifer J The intestinal microbiota affect central levels of brain-derived neurotropic factor and behavior in mice. Gastroenterology. (2011) 141:599–609.e3. 10.1053/j.gastro.2011.04.052 21683077

[9] de Rijk MC Launer LJ Berger K Breteler MM Dartigues JF Baldereschi M. Prevalence of Parkinson's disease in Europe: a collaborative study of population-based cohorts. Neurologic diseases in the elderly research group. Neurology. (2000) 54(11 Suppl. 5):21–3. 10.1212/WNL.54.11.21A 10854357

[10] Aronson MK Alzheimer's disease and Parkinson's disease — NEJM. N Eng J Med. (2003) 348:1356–64. 10.1056/NEJM2003ra020003 12672864

[11] Perl DP Olanow CW Calne D. Alzheimer's disease and Parkinson's disease: distinct entities or extremes of a spectrum of neurodegeneration? Ann Neurol. (1998) 44(3Suppl.1):S19–31. 10.1002/ana.410440705 9749570

[12] Wolters EC Braak H Parkinson's disease: premotor clinico-pathological correlations. J Neural Transm Suppl. (2006) 70:309–19. 10.1007/978-3-211-45295-0_47 17017546

[13] Sulzer D. Multiple hit hypotheses for dopamine neuron loss in Parkinson's disease. Trends Neurosci. (2007) 30:244–50. 10.1016/j.tins.2007.03.009 17418429

[14] St Louis EK Boeve AR Boeve BF. REM Sleep Behavior Disorder in Parkinson's disease and other synucleinopathies. Mov Disord. (2017) 32:645–58. 10.1002/mds.27018 28513079

[15] Antje H Thomas H Cornelia H Ulrike S Susann J Heinz R Olfactory loss may be a first sign of idiopathic Parkinson's disease. Mov Disord. (2010) 22:839–42. 10.1002/mds.21413 17357143

[16] Ponsen MM Diederick S Twisk JWR Erik Ch W Berendse HW. Hyposmia and executive dysfunction as predictors of future Parkinson's disease: a prospective study. Mov Disord. (2010) 24:1060–5. 10.1002/mds.22534 19353591

[17] Aarsland D Creese B Politis M Chaudhuri KR Ffytche DH Weintraub D. Cognitive decline in Parkinson disease. Nat Rev Neurol. (2017) 13:217–31. 10.1038/nrneurol.2017.27 28257128 PMC5643027

[18] Lim SY Lang AE. The nonmotor symptoms of Parkinson's disease&mdash;An overview. Mov Disord. (2010) 25(Suppl. 1):S123–30. 10.1002/mds.22786 20187234

[19] Claudio R Laura P Colonic mucosal α-synuclein lacks specificity as a biomarker for Parkinson disease. Neurology. (2015) 85:609–16. 10.1212/WNL.0000000000001904 PMC4335990 25589666

[20] Edwards LL Quigley EM Pfeiffer RF Gastrointestinal dysfunction in Parkinson's disease: frequency and pathophysiology. Semin Neurol. (2011) 17:10–5. 10.1212/wnl.42.4.726 1565224

[21] Savica R Carlin JM Grossardt BR Bower JH Ahlskog JE Maraganore DM. Medical records documentation of constipation preceding Parkinson disease: a case-control study. Neurology. (2009) 73:1752–8. 10.1212/WNL.0b013e3181c34af5 19933976 PMC2788809

[22] Abbott RD Petrovitch H Masaki KH Tanner CM Curb JD Grandinetti A. Frequency of bowel movements and the future risk of Parkinson's disease. Neurology. (2001) 57:456–62. 10.1212/WNL.57.3.456 11502913

[23] Chen H Zhao EJ Zhang W Lu Y Liu R Huang X. Meta-analyses on prevalence of selected Parkinson's nonmotor symptoms before and after diagnosis. Transl Neurodegener. (2015) 4:1. 10.1186/2047-9158-4-1 25671103 PMC4322463

[24] Egberto Reis B Non-motor symptoms in Parkinson's disease. Int J Neurosci. (2013) 121(Suppl. 2):9–17. 10.3109/00207454.2011.620196 22035025

[25] Braak H Vos RAID Bohl J Tredici KD. Gastric alpha-synuclein immunoreactive inclusions in Meissner's and Auerbach's plexuses in cases staged for Parkinson's disease-related brain pathology. Neurosci Lett. (2006) 396:67–72. 10.1016/j.neulet.2005.11.012 16330147

[26] Shannon KM Keshavarzian A Dodiya HB Jakate S Kordower JH. Is alpha-synuclein in the colon a biomarker for premotor Parkinson's disease? Evidence from 3 cases. Mov Disord. (2012) 27:716–9. 10.1002/mds.25020 22550057

[27] Borghammer P. How does parkinson's disease begin? Perspectives on neuroanatomical pathways, prions, and histology. Mov Disord. (2017) 33:48–57. 10.1002/mds.27138 28843014

[28] Gershanik OS Gershanik OS. Does Parkinson's disease start in the gut? Arq Neuropsiquiatr. (2018) 76:67–70. 10.1590/0004-282x20170188 29489958

[29] Surmeier DJ Halliday GM Simuni T. Calcium, mitochondrial dysfunction and slowing the progression of Parkinson's disease. Exp Neurol. (2017) 298(Pt B):202–9. 10.1016/j.expneurol.2017.08.001 28780195 PMC6037988

[30] Edwards LL Quigley EMM Pfeiffer RF. Gastrointestinal dysfunction in Parkinson's disease. Neurology. (1992) 42:726–32. 10.1212/WNL.42.4.726 1565224

[31] Oyanagi K Wakabayashi K Ohama E Takeda S Horikawa Y Morita T. Lewy bodies in the lower sacral parasympathetic neurons of a patient with Parkinson's disease. Acta Neuropathologica. (1990) 80:558–9. 10.1007/BF00294619 2251914

[32] Qualman SJ Haupt HM Yang P Hamilton SR. Esophageal Lewy bodies associated with ganglion cell loss in achalasia. Similarity to Parkinson's disease. Gastroenterology. (1984) 87:848–56. 10.1016/0016-5085(84)90079-9 6088351

[33] Wakabayashi K Takahashi H Takeda S Ohama E Ikuta F. Parkinson's disease: the presence of Lewy bodies in Auerbach's and Meissner's plexuses. Acta Neuropathologica. (1988) 76:217–21. 10.1007/BF00687767 2850698

[34] Braak H Del Tredici K Rub U de Vos RA Jansen Steur EN Braak E. Staging of brain pathology related to sporadic Parkinson's disease. Neurobiol Aging. (2003) 24:197–211. 10.1016/S0197-4580(02)00065-9 12498954

[35] Braak H Rüb U Gai WP Del Tredici K. Idiopathic Parkinson's disease: possible routes by which vulnerable neuronal types may be subject to neuroinvasion by an unknown pathogen. J Neural Transm. (2003) 110:517–36. 10.1007/s00702-002-0808-2 12721813

[36] Forsyth CB Shannon KM Kordower JH Voigt RM Shaikh M Jaglin JA. Increased intestinal permeability correlates with sigmoid mucosa alpha-synuclein staining and endotoxin exposure markers in early Parkinson's disease. PLoS ONE. (2011) 6:e28032. 10.1371/journal.pone.0028032 22145021 PMC3228722

[37] Phillips RJ Walter GC Wilder SL Baronowsky EA Powley TL. Alpha-synuclein-immunopositive myenteric neurons and vagal preganglionic terminals: autonomic pathway implicated in Parkinson's disease? Neuroscience. (2008) 153:733–50. 10.1016/j.neuroscience.2008.02.074 18407422 PMC2605676

[38] Sudo N Chida Y Aiba Y Sonoda J Oyama N Yu XN. Postnatal microbial colonization programs the hypothalamic-pituitary-adrenal system for stress response in mice. J Physiol. (2004) 558(Pt 1):263–75. 10.1113/jphysiol.2004.063388 15133062 PMC1664925

[39] Mccorry LK. Physiology of the autonomic nervous system. Am J Pharm Educ. (2007) 71:17–20. 10.5688/aj710478 17786266 PMC1959222

[40] Tsigos C Chrousos GP. Hypothalamic-pituitary-adrenal axis, neuroendocrine factors and stress. J Psychosom Res. (2002) 53:865–71. 10.1016/S0022-3999(02)00429-4 12377295

[41] Lilly MP Gann DS. The hypothalamic-pituitary-adrenal-immune axis. A critical assessment. Arch Surg. (1992) 127:1463–74. 10.1001/archsurg.1992.01420120097017 1365694

[42] Schemann M Neunlist M. The human enteric nervous system. Neurogastroenterol Motil. (2010) 16(Suppl. 1):55–9. 10.1111/j.1743-3150.2004.00476.x 15066006

[43] Jessen KR Mirsky R. Astrocyte-like glia in the peripheral nervous system: an immunohistochemical study of enteric glia. J Neurosci. (1983) 3:2206–18. 10.1523/JNEUROSCI.03-11-02206.1983 6138397 PMC6564643

[44] Gribble FM Reimann F. Enteroendocrine Cells: chemosensors in the intestinal epithelium. Ann Rev Physiol. (2016) 78:277–99. 10.1146/annurev-physiol-021115-105439 26442437

[45] Marchiando AM Graham WV Turner JR. Epithelial barriers in homeostasis and disease. Annu Rev Pathol. (2010) 5:119–44. 10.1146/annurev.pathol.4.110807.092135 20078218

[46] Suzuki T. Regulation of intestinal epithelial permeability by tight junctions. Cell Mol Life Sci. (2013) 70:631–59. 10.1007/s00018-012-1070-x 22782113 PMC11113843

[47] Bhattarai Y. Microbiota-gut-brain axis: interaction of gut microbes and their metabolites with host epithelial barriers. Neurogastroenterol Motil. (2018) 30:e13366. 10.1111/nmo.13366 29878576

[48] Hawkes CH Del Tredici K Braak H. Parkinson's disease: the dual hit theory revisited. Ann N Y Acad Sci. (2009) 1170:615–22. 10.1111/j.1749-6632.2009.04365.x 19686202

[49] Hawkes CH Del Tredici K Braak H. A timeline for Parkinson's disease. Parkinsonism Relat Disord. (2010) 16:79–84. 10.1016/j.parkreldis.2009.08.007 19846332

[50] Panmontojo FJ Anichtchik O Dening Y Knells L Pursche S Jung R Progression of Parkinson's disease pathology is reproduced by intragastric administration of rotenone in mice. PLoS ONE. (2010) 5:e8762 10.1371/journal.pone.0008762 20098733 PMC2808242

[51] Fasano A Visanji NP Liu LW Lang AE Pfeiffer RF. Gastrointestinal dysfunction in Parkinson's disease. Lancet Neurol. (2015) 14:625–39. 10.1016/S1474-4422(15)00007-1 25987282

[52] Holmqvist S Chutna O Bousset L Aldrinkirk P Li W Björklund T. Direct evidence of Parkinson pathology spread from the gastrointestinal tract to the brain in rats. Acta Neuropathologica. (2014) 128:805–20. 10.1007/s00401-014-1343-6 25296989

[53] Liu B Fang F Pedersen NL Tillander A Ludvigsson JF Ekbom A. Vagotomy and Parkinson disease: a Swedish register–based matched-cohort study. Neurology. (2017) 88:1996–2002. 10.1212/WNL.0000000000003961 28446653 PMC5440238

[54] Stokholm MG Danielsen EH Hamilton-Dutoit SJ Borghammer P. Pathological α-synuclein in gastrointestinal tissues from prodromal Parkinson disease patients. Anna Neurol. (2016) 79:940–9. 10.1002/ana.24648 27015771

[55] Bohórquez DV Samsa LA Roholt A Medicetty S Chandra R Liddle RA. An enteroendocrine cell-enteric glia connection revealed by 3D electron microscopy. PLoS ONE. (2014) 9:e89881. 10.1371/journal.pone.0089881 24587096 PMC3935946

[56] Bohórquez DV Shahid RA Erdmann A Kreger AM Wang Y Calakos N. Neuroepithelial circuit formed by innervation of sensory enteroendocrine cells. J Clin Invest. (2015) 125:782–6. 10.1172/JCI78361 25555217 PMC4319442

[57] Chandra R Hiniker A Kuo YM Nussbaum RL Liddle RA. α-Synuclein in gut endocrine cells and its implications for Parkinson's disease. JCI Insight. (2017) 2:92295. 10.1172/jci.insight.92295 28614796 PMC5470880

[58] Clairembault T Leclair-Visonneau L Coron E Bourreille A Le Dily S Vavasseur F. Structural alterations of the intestinal epithelial barrier in Parkinson's disease. Acta Neuropathol Commun. (2015) 3:12. 10.1186/s40478-015-0196-0 25775153 PMC4353469

[59] Ha CW Lam YY Holmes AJ. Mechanistic links between gut microbial community dynamics, microbial functions and metabolic health. World J Gastroenterol. (2014) 20:16498–517. 10.3748/wjg.v20.i44.16498 25469018 PMC4248193

[60] Levy M Kolodziejczyk AA Thaiss CA Elinav EJNRI. Dysbiosis and the immune system. Nat Rev Immunol. (2017) 17:219–32. 10.1038/nri.2017.7 28260787

[61] Houser MC Tansey MG. The gut-brain axis: is intestinal inflammation a silent driver of Parkinson's disease pathogenesis? NPJ Parkinsons Dis. (2017) 3:3. 10.1038/s41531-016-0002-0 28649603 PMC5445611

[62] Houser MC Chang J Factor SA Molho ES Zabetian CP Hill-Burns EM. Stool immune profiles evince gastrointestinal inflammation in Parkinson's disease. Mov Disord. (2018) 33:793–804. 10.1002/mds.27326 29572994 PMC5992021

[63] Kelly LP Carvey PM Keshavarzian A Shannon KM Shaikh M Bakay RA. Progression of intestinal permeability changes and alpha-synuclein expression in a mouse model of Parkinson's disease. Mov Disord. (2014) 29:999–1009. 10.1002/mds.25736 24898698 PMC4050039

[64] Mukherjee A Biswas A Das SK. Gut dysfunction in Parkinson's disease. World J Gastroenterol. (2016) 22:5742–52. 10.3748/wjg.v22.i25.5742 27433087 PMC4932209

[65] Keshavarzian A Green SJ Engen PA Voigt RM Naqib A Forsyth CB. Colonic bacterial composition in Parkinson's disease. Mov Disor. (2015) 30:1351–60. 10.1002/mds.26307 26179554

[66] Hasegawa S Goto S Tsuji H Okuno T Asahara T Nomoto K. Intestinal dysbiosis and lowered serum lipopolysaccharide-binding protein in Parkinson's disease. PLoS ONE. (2015) 10:e0142164. 10.1371/journal.pone.0142164 26539989 PMC4634857

[67] Devos D Lebouvier T Lardeux B Biraud M Rouaud T Pouclet H. Colonic inflammation in Parkinson's disease. Neurobiol Dis. (2013) 50:42–8. 10.1016/j.nbd.2012.09.007 23017648

[68] Bialecka M Kurzawski M Klodowska-Duda G Opala G Juzwiak S Kurzawski G. CARD15 variants in patients with sporadic Parkinson's disease. (2007) 57:473–6. 10.1016/j.neures.2006.11.012 17174426

[69] Liu JZ van Sommeren S Huang H Ng SC Alberts R Takahashi A. Association analyses identify 38 susceptibility loci for inflammatory bowel disease and highlight shared genetic risk across populations. Nat Genet. (2015) 47:979–86. 10.1038/ng.3359 26192919 PMC4881818

[70] Hui KY Fernandez-Hernandez H Hu J Schaffner A Pankratz N Hsu NY. Functional variants in the LRRK2 gene confer shared effects on risk for Crohn's disease and Parkinson's disease. Sci Transl Med. (2018) 10:eaai7795. 10.1126/scitranslmed.aai7795 29321258 PMC6028002

[71] Clairembault T Kamphuis W Leclair-Visonneau L Rolli-Derkinderen M Coron E Neunlist M. Enteric GFAP expression and phosphorylation in Parkinson's disease. J Neurochem. (2014) 130:805–15. 10.1111/jnc.12742 24749759

[72] Carolina P Matteo F Rocchina C Erika T Fabio B Giovanna L Alteration of colonic excitatory tachykininergic motility and enteric inflammation following dopaminergic nigrostriatal neurodegeneration. J Neuroinflammation. (2016) 13:1–13. 10.1186/s12974-016-0608-5 27295950 PMC4907252

[73] Houser MC Chang J Factor SA Molho ES Zabetian CP Hillburns EM Stool immune profiles evince gastrointestinal inflammation in Parkinson's disease. Neurobiol Dis. (1997) 30:125–43. 10.1002/mds.27326 PMC5992021 29572994

[74] Louveau A Smirnov I Keyes TJ Eccles JD Rouhani SJ Peske JD. Structural and functional features of central nervous system lymphatic vessels. Nature. (2016) 523:337–41. 10.1038/nature14432 26030524 PMC4506234

[75] Takeuchi O Akira SJC. Pattern recognition receptors and inflammation. Cell. (2010) 140:805–20. 10.1016/j.cell.2010.01.022 20303872

[76] Friedland RP. Mechanisms of molecular mimicry involving the microbiota in neurodegeneration. J Alzheimers Dis. (2015) 45:349–62. 10.3233/JAD-142841 25589730

[77] Schwartz K Boles BR. Microbial amyloids – functions and interactions within the host. Curr Opin Microbiol. (2013) 16:93–9. 10.1016/j.mib.2012.12.001 23313395 PMC3622111

[78] Biagi E Candela M Turroni S Garagnani P Franceschi C Brigidi PJPRtOJotIPS. Ageing and gut microbes: perspectives for health maintenance and longevity. Pharmacol Res. (2013) 69:11–20. 10.1016/j.phrs.2012.10.005 23079287

[79] Glass CK Saijo K Winner B Marchetto MC Gage FH. Mechanisms underlying inflammation in neurodegeneration. Cell. (2010) 140:918–34. 10.1016/j.cell.2010.02.016 20303880 PMC2873093

[80] Quigley EM Quera R. Small intestinal bacterial overgrowth: roles of antibiotics, prebiotics, and probiotics. Gastroenterology. (2006) 130:S78–90. 10.1053/j.gastro.2005.11.046 16473077

[81] Çamci G Oguz S. Association between Parkinson's disease and *Helicobacter Pylori*. J Clin Neurol. (2016) 12:147–50. 10.3988/jcn.2016.12.2.147 26932258 PMC4828559

[82] Alfonso Fasano MD Francesco Bove MD Maurizio Gabrielli MD Martina Petracca MD Zocco MA Cbc ER. The role of small intestinal bacterial overgrowth in Parkinson's disease. Mov Disord. (2013) 28:1241–9. 10.1002/mds.25522 23712625

[83] Tan AH Mahadeva S Thalha AM Gibson PR Kiew CK Yeat CM. Small intestinal bacterial overgrowth in Parkinson's disease. Parkinsonism Relat Disord. (2014) 20:535–40. 10.1016/j.parkreldis.2014.02.019 24637123

[84] Scheperjans F Aho V Pereira PA Koskinen K Paulin L Pekkonen E. Gut microbiota are related to Parkinson's disease and clinical phenotype. Mov Disord. (2015) 30:350–8. 10.1002/mds.26069 25476529

[85] Sampson TR Debelius JW Thron T Janssen S Shastri GG Ilhan ZE. Gut microbiota regulate motor deficits and neuroinflammation in a model of Parkinson's disease. Cell. (2016) 167:1469–80.e12. 10.1016/j.cell.2016.11.018 27912057 PMC5718049

[86] Hill-Burns EM Debelius JW Morton JT Wissemann WT Lewis MR Wallen ZD. Parkinson's disease and Parkinson's disease medications have distinct signatures of the gut microbiome. Mov Disord. (2017) 32:739–49. 10.1002/mds.26942 28195358 PMC5469442

[87] Arumugam M Raes J Pelletier E Paslier DL Yamada T Mende DR Addendum: enterotypes of the human gut microbiome. Nature. (2011) 473:174–80. 10.1038/nature09944 21508958 PMC3728647

[88] Andrews ZB Erion D Beiler R. Ghrelin promotes and protects nigrostriatal dopamine function via a UCP2-dependent mitochondrial mechanism. J Neurosci. (2009) 29:14057–65. 10.1523/JNEUROSCI.3890-09.2009 19906954 PMC2845822

[89] Unger MM Möller JC Mankel K Schmittinger K Eggert KM Stamelou M. Patients with idiopathic rapid-eye-movement sleep behavior disorder show normal gastric motility assessed by the 13C-octanoate breath test. Mov Disord. (2011) 26:2559–63. 10.1002/mds.23933 22147682

[90] Hueck CJ. Type III protein secretion systems in bacterial pathogens of animals and plants. Microbiol Mol Biol Rev. (1998) 62:379–433. 9618447 10.1128/mmbr.62.2.379-433.1998 PMC98920

[91] Galan JE Collmer A. Type III secretion machines: bacterial devices for protein delivery into host cells. Science. (1999) 284:1322–8. 10.1126/science.284.5418.1322 10334981

[92] Unger MM Spiegel J Dillmann KU Grundmann D Philippeit H Bürmann J. Short chain fatty acids and gut microbiota differ between patients with Parkinson's disease and age-matched controls. Parkinsonism Relat Disord. (2016) 32:66–72. 10.1016/j.parkreldis.2016.08.019 27591074

[93] Ganapathy V Thangaraju M Prasad PD Martin PM Singh N. Transporters and receptors for short-chain fatty acids as the molecular link between colonic bacteria and the host. Curr Opin Pharmacol. (2013) 13:869–74. 10.1016/j.coph.2013.08.006 23978504

[94] Maslowski KM Mackay CR. Diet, gut microbiota and immune responses. Nat Immunol. (2011) 12:5–9. 10.1038/ni0111-5 21169997

[95] Singh N Gurav A Sivaprakasam S Brady E Padia R Shi H. Activation of Gpr109a, receptor for niacin and the commensal metabolite butyrate, suppresses colonic inflammation and carcinogenesis. Immunity. (2014) 40:128–39. 10.1016/j.immuni.2013.12.007 24412617 PMC4305274

[96] Derkinderen P Shannon KM Brundin P. Gut feelings about smoking and coffee in Parkinson's disease. Mov Disord. (2014) 29:976–9. 10.1002/mds.25882 24753353 PMC4107006

[97] Minato T Maeda T Fujisawa Y Tsuji H Nomoto K Ohno K. Progression of Parkinson's disease is associated with gut dysbiosis: two-year follow-up study. PLoS ONE. (2017) 12:e0187307. 10.1371/journal.pone.0187307 29091972 PMC5665539

[98] Bested AC Logan AC Selhub EM. Intestinal microbiota, probiotics and mental health: from Metchnikoff to modern advances: part I - autointoxication revisited. Gut Pathog. (2013) 5:5. 10.1186/1757-4749-5-5 23506618 PMC3607857

[99] Wall R Cryan JF Ross RP Fitzgerald GF Dinan TG Stanton C Bacterial Neuroactive Compounds Produced by Psychobiotics Adv Exp Med Biol. (2014) 817:221–39. 10.1007/978-1-4939-0897-4_10 24997036

[100] Eisenhofer G Åneman A Friberg P Hooper D Fåndriks L Lonroth H. Substantial production of dopamine in the human gastrointestinal tract. J Clin Endocrinol Metab. (1997) 82:3864–71. 10.1210/jcem.82.11.4339 9360553

[101] Crispo JAG Fortin Y Thibault DP Emons M Bjerre LM Kohen DE. Trends in inpatient antiparkinson drug use in the USA, 2001–2012. Eur J Clin Pharmacol. (2015) 71:1011–9. 10.1007/s00228-015-1881-4 26081062 PMC4500853

[102] Lee HM Koh SB. Many faces of Parkinson's disease: non-motor symptoms of Parkinson's disease. J Mov Disord. (2015) 8:92–7. 10.14802/jmd.15003 26090081 PMC4460545

[103] Lebouvier T Neunlist M Bruley des Varannes S Coron E Drouard A N'Guyen JM. Colonic biopsies to assess the neuropathology of Parkinson's disease and its relationship with symptoms. PLoS ONE. (2010) 5:e12728. 10.1371/journal.pone.0012728 20856865 PMC2939055

[104] O'Neill C. Gut microbes metabolize Parkinson's disease drug. Science. (2019) 364:1030–1. 10.1126/science.aax8937 31196998

[105] Tambasco N Romoli M Calabresi P. Levodopa in Parkinson's disease: current status and future developments. Curr Neuropharmacol. (2018) 16:1239–52. 10.2174/1570159X15666170510143821 28494719 PMC6187751

[106] Perez-Pardo P de Jong EM Broersen LM van Wijk N Attali A Garssen J. Promising effects of neurorestorative diets on motor, cognitive, and gastrointestinal dysfunction after symptom development in a mouse model of Parkinson's disease. Front Aging Neurosci. (2017) 9:57. 10.3389/fnagi.2017.00057 28373840 PMC5357625

[107] Reid G Younes JA Van der Mei HC Gloor GB Knight R Busscher HJ. Microbiota restoration: natural and supplemented recovery of human microbial communities. Nat Rev Microbiol. (2011) 9:27–38. 10.1038/nrmicro2473 21113182

[108] Varankovich NV Nickerson MT Korber DR. Probiotic-based strategies for therapeutic and prophylactic use against multiple gastrointestinal diseases. Front Microbiol. (2015) 6:685. 10.3389/fmicb.2015.00685 26236287 PMC4500982

[109] Rao AV Bested AC Beaulne TM Katzman MA Iorio C Berardi JM. A randomized, double-blind, placebo-controlled pilot study of a probiotic in emotional symptoms of chronic fatigue syndrome. Gut Pathog. (2009) 1:6. 10.1186/1757-4749-1-6 19338686 PMC2664325

[110] Liang S Wang T Hu X Luo J Li W Wu X. Administration of Lactobacillus helveticus NS8 improves behavioral, cognitive, and biochemical aberrations caused by chronic restraint stress. Neuroscience. (2015) 310:561–77. 10.1016/j.neuroscience.2015.09.033 26408987

[111] Gibson GR Roberfroid MB. Dietary modulation of the human colonic microbiota: introducing the concept of prebiotics. J Nutr. (1995) 125:1401–12. 10.1093/jn/125.6.1401 7782892

[112] Scheperjans F Pekkonen E Kaakkola S Auvinen P. Linking smoking, coffee, urate, and Parkinson's disease–a role for gut microbiota? J Parkinsons Dis. (2015) 5:255–62. 10.3233/JPD-150557 25882059

[113] Biedermann L Brülisauer K Zeitz J Frei P Scharl M Vavricka SR. Smoking cessation alters intestinal microbiota: insights from quantitative investigations on human fecal samples using FISH. Inflamm Bowel Dis. (2014) 20:1496–501. 10.1097/MIB.0000000000000129 25072500

[114] Mills CE Tzounis X Oruna-Concha MJ Mottram DS Gibson GR Spencer JP. *In vitro* colonic metabolism of coffee and chlorogenic acid results in selective changes in human faecal microbiota growth. Br J Nutr. (2015) 113:1220–7. 10.1017/S0007114514003948 25809126

[115] Dodiya HB Forsyth CB Voigt RM Engen PA Patel J Shaikh M. Chronic stress-induced gut dysfunction exacerbates Parkinson's disease phenotype and pathology in a rotenone-induced mouse model of Parkinson's disease. Neurobiol Dis. (2018). [Epub ahead of print]. 10.1016/j.nbd.2018.12.012 30579705

[116] Fava F Gitau R Griffin BA Gibson GR Tuohy KM Lovegrove JA. The type and quantity of dietary fat and carbohydrate alter faecal microbiome and short-chain fatty acid excretion in a metabolic syndrome 'at-risk' population. Int J Obes. (2013) 37:216–23. 10.1038/ijo.2012.33 22410962

[117] Ascherio A Schwarzschild MA. The epidemiology of Parkinson's disease: risk factors and prevention. Lancet Neurol. (2016) 15:1257–72. 10.1016/S1474-4422(16)30230-7 27751556

[118] Monda V Villano I Messina A Valenzano A Esposito T Moscatelli F. Exercise Modifies the Gut Microbiota with Positive Health Effects. Oxid Med Cell Longev. (2017) 2017:3831972. 10.1155/2017/3831972 28357027 PMC5357536

[119] Estaki M Pither J Baumeister P Little JP Gill SK Ghosh S. Cardiorespiratory fitness as a predictor of intestinal microbial diversity and distinct metagenomic functions. Microbiome. (2016) 4:42. 10.1186/s40168-016-0189-7 27502158 PMC4976518

[120] Davey KJ Cotter PD O'Sullivan O Crispie F Dinan TG Cryan JF. Antipsychotics and the gut microbiome: olanzapine-induced metabolic dysfunction is attenuated by antibiotic administration in the rat. Transl Psychiatry. (2013) 3:e309. 10.1038/tp.2013.83 24084940 PMC3818006

[121] Parashar A Udayabanu M. Gut microbiota: Implications in Parkinson's disease. Parkinsonism Relat Disord. (2017) 38:1–7. 10.1016/j.parkreldis.2017.02.002 28202372 PMC7108450

[122] Zhang F Luo W Shi Y Fan Z Ji G. Should we standardize the 1,700-year-old fecal microbiota transplantation? Am J Gastroenterol. (2012) 107:1755. author reply p. 1755–6. 10.1038/ajg.2012.251 23160295

[123] Aroniadis OC Brandt LJ. Fecal microbiota transplantation: past, present and future. Med J Aust. (2013) 29:79–84. 10.1097/MOG.0b013e32835a4b3e 23041678

[124] Borody TJ Khoruts A. Fecal microbiota transplantation and emerging applications. Nat Rev Gastroenterol Hepatol. (2012) 9:88–96. 10.1038/nrgastro.2011.244 22183182

[125] Collins SM Surette M Bercik P. The interplay between the intestinal microbiota and the brain. Nat Rev Microbiol. (2012) 10:735–42. 10.1038/nrmicro2876 23000955

[126] Sun MF Zhu YL Zhou ZL Jia XB Xu YD Yang Q. Neuroprotective effects of fecal microbiota transplantation on MPTP-induced Parkinson's disease mice: Gut microbiota, glial reaction and TLR4/TNF-alpha signaling pathway. Brain Behav Immun. (2018) 70:48–60. 10.1016/j.bbi.2018.02.005 29471030

[127] Borody TJ Debra P Mitchell SW. Fecal microbiota transplantation: expanding horizons for clostridium difficile infections and beyond. Antibiotics. (2015) 4:254–66. 10.3390/antibiotics4030254 27025624 PMC4790284

[128] de Vos WM dev Vos EA. Role of the intestinal microbiome in health and disease: from correlation to causation. Nutr Rev. (2012) 70(Suppl. 1):S45–56. 10.1111/j.1753-4887.2012.00505.x 22861807

[129] Lionnet A Leclair-Visonneau L Neunlist M Murayama S Takao M Adler CH. Does Parkinson's disease start in the gut? Acta Neuropathol. (2018) 135:1–12. 10.1007/s00401-017-1777-8 29039141

[130] Adler CH Beach TG. Neuropathological basis of nonmotor manifestations of Parkinson's disease. Mov Disord. (2016) 31:1114–9. 10.1002/mds.26605 27030013 PMC4981515

