# Corrigendum: The Role of the Gut Microbiota in the Pathogenesis of Parkinson's Disease

**DOI:** 10.3389/fneur.2019.01412

**Published:** 2020-02-06

**Authors:** Dongming Yang, Deming Zhao, Syed Zahid Ali Shah, Wei Wu, Mengyu Lai, Xixi Zhang, Jie Li, Zhiling Guan, Huafen Zhao, Wen Li, Hongli Gao, Xiangmei Zhou, Lifeng Yang

**Affiliations:** ^1^Key Laboratory of Animal Epidemiology and Zoonosis, Ministry of Agriculture, National Animal Transmissible Spongiform Encephalopathy Laboratory, College of Veterinary Medicine, China Agricultural University, Beijing, China; ^2^Department of Pathology, Faculty of Veterinary Sciences, Cholistan University of Veterinary and Animal Sciences, Bahawalpur, Pakistan

**Keywords:** parkinson's disease, gut microbiota, enteric nervous system, gut-brain axis, microbiota-targeted therapies, fecal transplant

In the original article there was an error with the **Author Contributions** statement. This statement was intended to be used in a different article, also written by the authors.

A correction has therefore been made to the **Author Contributions** statement:

“DY wrote the first draft of the manuscript. DZ and LY revised the initial drafts and provided scientific contributions. All remaining authors edited the final version of the manuscript.”

The authors apologize for this error and state that this does not change the scientific conclusions of the article in any way. The original article has been updated.

